# Analysis of the Overall Structure of the Multi-Domain Amyloid Precursor Protein (APP)

**DOI:** 10.1371/journal.pone.0081926

**Published:** 2013-12-04

**Authors:** Ina Coburger, Sven O. Dahms, Dirk Roeser, Karl-Heinz Gührs, Peter Hortschansky, Manuel E. Than

**Affiliations:** 1 Protein Crystallography Group, Leibniz Institute for Age Research - Fritz Lipmann Institute (FLI), Jena, Germany; 2 Protein Laboratory, Leibniz Institute for Age Research - Fritz Lipmann Institute (FLI), Jena, Germany; 3 Molecular and Applied Microbiology, Leibniz Institute for Natural Product Research and Infection Biology - Hans-Knöll-Institute (HKI), Jena, Germany; Cleveland Clnic Foundation, United States of America

## Abstract

The amyloid precursor protein (APP) and its processing by the α-, β- and γ-secretases is widely believed to play a central role during the development of Alzheimer´s disease. The three-dimensional structure of the entire protein, its physiologic function and the regulation of its proteolytic processing remain, however, largely unclear to date. To gain a deeper understanding of the structure of APP that underlies all of its functions, we first cloned and recombinantly expressed different constructs in *E. coli*. Using limited proteolysis followed by mass spectrometry and Edman degradation as well as analytical gel permeation chromatography coupled static light scattering, we experimentally analyzed the structural domain boundaries and determined that the large ectodomain of APP consists of exactly two rigidly folded domains – the E1-domain (Leu18-Ala190) and the E2-domain (Ser295-Asp500). Both, the acidic domain (AcD) connecting E1 and E2 as well as the juxtamembrane region (JMR) connecting E2 to the single transmembrane helix are highly flexible and extended. We identified in-between the E1-domain and the AcD an additional domain of conservation and partial flexibility that we termed extension domain (ED, Glu191-Glu227). Using Bio-layer interferometry, pull-down assays and analytical gel filtration experiments we demonstrated that the E1-domain does not tightly interact with the E2-domain, both in the presence and in the absence of heparin. APP hence forms an extended molecule that is flexibly tethered to the membrane. Its multi-domain architecture enables together with the many known functionalities the concomitant performance of several, independent functions, which might be regulated by cellular, compartment specific pH-changes.

## Introduction

Alzheimer´s disease (AD) is one of the most common forms of dementia worldwide. One important role plays the amyloid precursor protein (APP) - a type I transmembrane protein that is expressed in a wide range of different cell types including neurons [1,2] and belongs to a small gene family of APP-like proteins including APLP1 and APLP2 [3]. APP can be proteolytically processed by β- and γ-secretase, which leads to the generation of 38-43 amino acid long peptides (amyloidogenic pathway). Deposition of these Aβ-peptides as amyloid plaques in the brain is one of the immuno-pathological hallmarks of AD. Alternatively, initiation of the proteolysis cascade by α-secretase prevents the development of these toxic peptides due to a cleavage within the Aβ-sequence (non-amyloidogenic pathway) [4,5]. 

**Figure 1:**
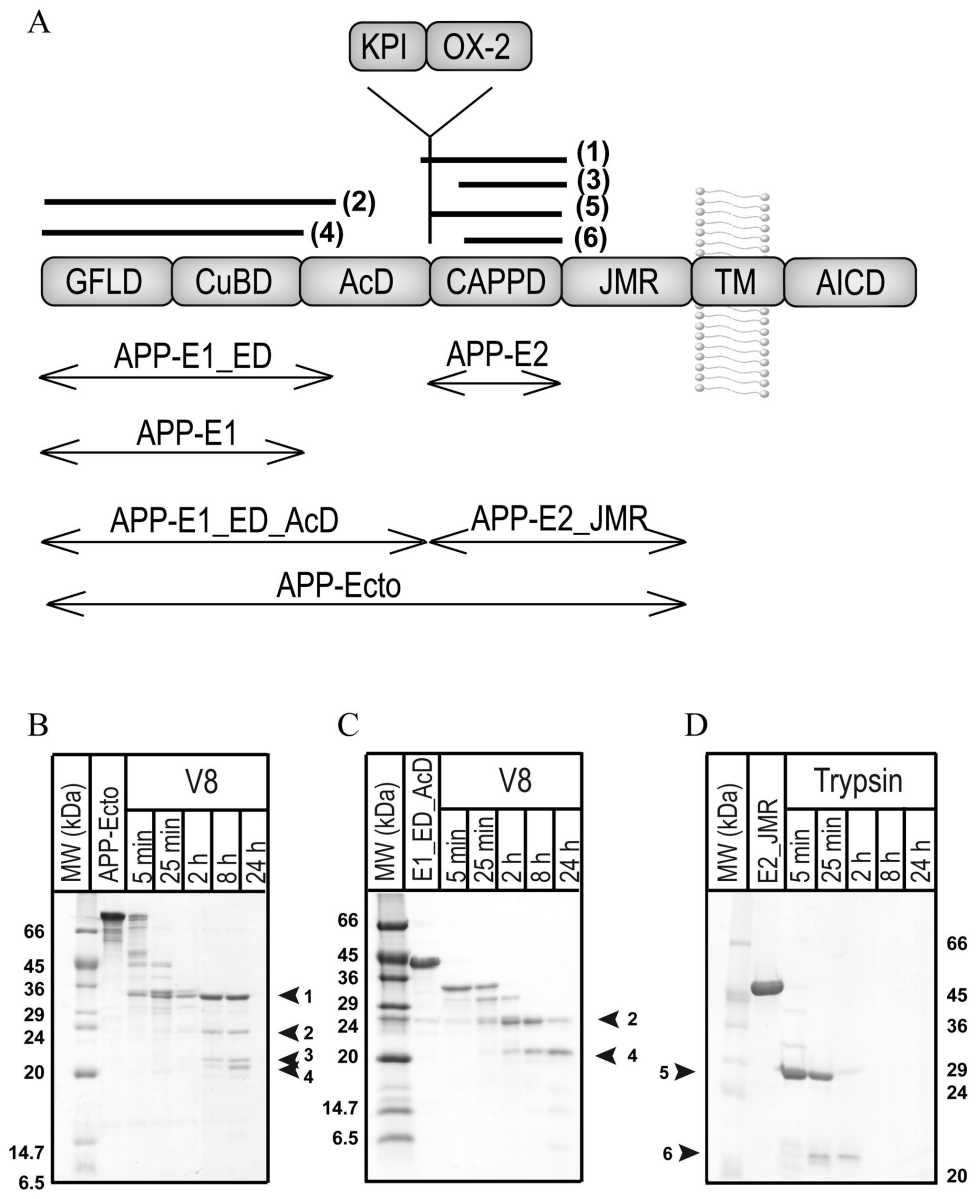
Domain architecture of the amyloid precursor protein (APP). (a) The ectodomain of APP consists of several subdomains or functional regions that are typically termed: the growth-factor-like-domain (GFLD), the copper-binding-domain (CuBD), the acidic domain (AcD), the central APP domain (CAPPD) and the juxtamembrane region (JMR) succeeded by a single transmembrane helix (TM) and the intracellular domain of APP (AICD). The longer splicing forms of APP, APP_751_ and APP_770_ contain additional regions, the Kunitz-type-inhibitor (KPI) domain and the Ox-2 sequence. Used constructs and limited proteolysis products of Table 1 are illustrated by double-headed arrows and black bars, respectively. (b-d) Limited proteolysis experiments with V8 protease and trypsin, respectively, demonstrate the flexibility of the acidic and juxtamembrane region and show that the E1- as well as the E2-domains are rigidly folded. The respective proteolysis conditions are given on top and the sizes of the molecular weight (MW) marker bands are given on the side of the gel in kDa. The cleavage sites and the sequence of the stable proteolysis fragments are listed in Table 1.

Because of its central role during the development of AD, APP and its proteolytic processing are in the focus of intensive research. Nevertheless, the physiologic function and the structure of the entire protein remain largely unclear until now. First insights into the domain architecture of APP could be obtained from homology considerations resulting in the initial definition of the E1- and the E2-domains within the large ectodomain [6,7,8]. Highly resolved structures of a number of individual domains of APP have been determined in the last ~20 years, such as those of its growth-factor-like domain (GFLD) [9], its copper-binding domain (CuBD) [10,11], its Kunitz-type protease inhibitor domain (KPI) (not present in the neuronal APP_695_ splice form) [12,13], the central APP domain (called CAPPD or E2-domain) [14,15,16] the structure of its membrane-proximal region [17] and that of its intracellular domain AICD [18,19]. In addition, the crystal structure of the entire E1-domain of APP shows that its constituting CuBD and GFLD interact tightly with one another, forming one rigid entity [20]. However, several domains, subdomains and functional segments are described in the literature with (largely) different and overlapping boundaries (Figure 1A). Those include e.g. the cysteine-rich domain, the GFLD, the CuBD, a heparin-binding domain, a zinc-binding domain and the acidic domain (AcD) within the N-terminal half of APP. Similarly, different domains are also specified for the C-terminal half of the large APP-ectodomain, including e.g. the central CAPPD, a second heparin-binding domain, the RERMS-domain, one collagen-binding domain and the juxtamembrane region (JMR) [21,22,23,24]. These protein segments must finally function within the currently unknown structural arrangement of the entire molecule. A first glimpse of the overall structure and into the arrangement of the different domains within the full-length protein could be obtained by small angle X-ray scattering (SAXS) experiments [25]. Nevertheless, there are only very limited data on the exact boundaries of folded segments and the interaction of the individual structural domains within the full-length protein. In particular, SAXS studies [25] [26] gave contradictory results with respect to an interaction of different domains. Investigating its overall structure and domain architecture will also answer the central question: whether APP represents one defined fold for its large ectodomain, or if its ectodomain must be considered rather as individual functional units that are flexibly connected to one another like pearls on a string. Additionally, more than one third of the APP-ectodomain consists of so far structurally uncharacterized regions, the AcD and the JMR, connecting E1 to E2 as well as E2 to the single transmembrane helix, respectively. 

In order to experimentally analyze the exact domain architecture of APP, to determine whether its constituting domains potentially interact with one another within the entire protein and to get a clear picture of the isolated APP, we employed herein a number of biochemical and biophysical methods investigating various recombinantly expressed APP-constructs.

## Materials and Methods

### Cloning

The plasmid pIK15 for expression of the entire APP-ectodomain (APP-Ecto, Leu18-Lys624, numbering according to APP_695_) inclusive a C-terminal His_6_-Tag was generated from pCK1 [27] by polymerase chain reaction (PCR) employing the primers 5´ AAAAAAAAAAAACATATGCTGGAGGTACCCACTG 3´ and 5´ AAAAAAAAAAGCTTACGACCTTCGATTTTGTTTGAACCCACATCTTCTGCAAAGAACAC 3´, followed by digestion with NdeI and HindIII and ligation into the vector pET22b(+).

The constructs encompassing APP-E2 and the juxtamembrane region (APP-E2_JMR, Ser295-Lys624) as well as the extended APP-E1 domain (APP-E1_ED, Leu18-Glu227) contain also a C-terminal His_6_-Tag and were generated by PCR from the template pCK1 using the primers 5´ AAAAAAAACATATGAGTACCCCTGATGCCGTTGACAAG 3´ and 5´ AAAAAAAA AAGCTTACGACCTTCGATTTTGTTTGAACCCACATCTTCTGCAAAGAACAC 3´, as well as 5´ AAAAAAAAAAAACATATGCTGGAGGTACCCACTG 3´ and 5´ AAAAAAAA GCTTACGACCTTCGATTTCTACTACTTTGTCTTCACTCCCATCTGCATAG 3´, respectively, followed by digestion and ligation as described above. 

The constructs APP-E1_ED_AcD (Leu18-R288), APP-E1 (Leu18-Ala190) and APP-E2 (Ser295-D500) were cloned as described before [15,20,27]. 

### Expression and Purification

APP-Ecto, APP-E1_ED_AcD and APP-E1_ED were expressed in *Escherichia coli* OrigamiB(*DE3*), whereas expression of APP-E2_JMR was performed in *E. coli* BL21(*DE3*)pRIL. Cells were grown in lysogeny broth (LB) at 25 °C, induced with 1 mM IPTG and harvested after 24 h by centrifugation at 4000 g for 20 min. 

The His_6_-tagged proteins were purified using a HisTrap FF Crude affinity column and a HiLoad Superdex 200 26/60 column. For APP-Ecto and APP-E1_ED an additional HiTrap Heparin column (all from GE Healthcare) was used. APP-E1 and APP-E2 were expressed and purified as described before [15,20]. For pull-down assays the C-terminal His_6_-tag was cleaved with 5 U/mg Factor Xa (Novagen) within 2 h at 20 °C. Uncleaved protein and Factor Xa were removed using a HisTrap FF Crude affinity column and a HiTrap Heparin column (GE Healthcare). 

### Sequence alignment

Sequences of APP were obtained from the UniProt (UP) database and aligned using the program ClustalX2 [28]: *Homo sapiens* APP (UP code: P05067-4), *Sus scofa* APP (UP code: P79307), *Bos Taurus* APP (UP code: Q08E54), *Gallus gallus* APP (UP code: F1NDE5), *Mus musculus* APP (UP code: P12023-2), *Rattus norvegicus* APP (UP code: P08592), *Xenopus laevis* APP (UP code: Q98SG0), *Danio rerio* APPa (UP code: Q6NUZ1), APPb (UP code: Q8UUR9). 

#### Limited Proteolysis

Limited proteolysis experiments were performed in 5 mM Tris pH 8.0, 150 mM NaCl containing 0.5 mg/ml protein and 50 µg/ml of the respective protease. The reactions were incubated at 25 °C and stopped after fixed time points using 10 mM PMSF. Samples were analyzed by SDS-PAGE. All limited proteolysis experiments were repeated in three independent experiments.

#### Edman-Sequencing

Limited proteolysis products were separated on a SDS gel and blotted onto a PVDF membrane. The membrane was stained with Coomassie and bands were analyzed using Edman degradation (Procise 494A, Applied Biosystems, Foster City, CA, USA). 

#### Mass spectrometry

Limited proteolysis samples were separated on a Superdex 200 5/150 GL column (GE Healthcare) and the total mass of the fractions were analyzed at the Center for Molecular Medicine Cologne (ZMMK, Central Bioanalytic, University of Cologne). In addition, protein containing fractions were precipitated with acetone. The pellet was resuspended in ammonium acetate (pH 7.5) and total mass was measured using Ultraflex II, Bruker Daltonics. 

### Calculation of the theoretical MW

The theoretical MW (MW_th_) was calculated using the ProtParam tool provided by ExPASy.

### GPC

Analytical size exclusion chromatography was performed in 5 mM Tris pH 8.0, 150 mM NaCl using a calibrated Superdex 200 5/150 GL column (GE Healthcare). All runs were repeated in three independent experiments. The column was calibrated using BSA, cytochrome c, carboanhydrase and aprotinin and a calibration curve was calculated using the molecular weight of the proteins as a function of the retention volume. The apparent molecular weight (MW_rh_) was determined using the retention volume of the protein and the calculated calibration curve. 

### GPC coupled SLS

For SLS measurements an Aekta Explorer system (GE Healthcare) was connected to a VE 3580 RI and 270 Dual detector (Viscotek). The absolute molecular weight (MW_SLS_) was determined using the OmniSEC software (Viscotek) provided with the instrument and based on the Rayleigh-Gans-Debye equation. All experiments were performed in 5 mM Tris pH 8.0, 150 mM NaCl using a Superdex 200 10/300 (GE-Healthcare) and were done in triplicate.

### CD spectroscopy

CD spectra were measured using a J-710 spectropolarimeter (JASCO Corporation) in 5 mM sodium phosphate buffer pH 7.5. Resulting data were analyzed using Spectra Analysis and CD Spectra Deconvolution 2.1 (JASCO Corporation). All measurements were repeated in three independent experiment. 

### Pull-down Assay

Pull-down experiments were performed in binding buffer (5 mM Tris pH 8.0, 150 mM NaCl, 20 mM imidazole, 0.05 % Tween20) using 80 µl Ni-NTA material (Qiagen). 5 µM His-tagged protein were incubated with 5 µM protein without His_6_-tag at 8 °C for 2 h. Where applicable 50 µM short chain heparin (low molecular weight heparin sodium salt, Sigma-Aldrich; corresponding to 10-12 sugar rings) or long chain heparin (heparin sodium salt, Sigma-Aldrich; corresponding to ~55 sugar rings) was added to the solution. Samples were centrifuged at 500xg for 1 min and washed with binding buffer. To analyze bound proteins, the beads were mixed with 2x sample buffer (0.15 M Tris/HCl pH 6.8, 1.2 % SDS, 30 % glycerol, 15 % mercaptoethanol and a small amount of bromophenol blue), incubated at 95 °C for 5 min and samples were analyzed by SDS-PAGE. All experiments were performed in triplicate.

### Bio-layer interferometry

Interaction analysis between APP-E1_ED_AcD and APP-E2_JMR domains was performed on an Octet RED96 instrument (ForteBio) at 28 °C. Biotinylated APP-E1_ED_AcD was prepared by incubating APP-E1_ED_AcD (5 µM) with Sulfo-NHS-LC-Biotin (Thermo) at a molar ratio of 1:1 for 3 hours at 4 °C in PBS, followed by desalting using a PD MiniTrap G25 column (GE Healthcare) to remove the excess biotin reagent. A column of eight Streptavidin biosensor tips were loaded with biotinylated APP-E1_ED_AcD (0.2 µM) in 1x kinetics buffer (10 mM phosphate, 2.7 mM KCl, 137 mM NaCl (pH 7.4) containing 0.1 mg/ml BSA and 0.002% (v/v) Tween20) to a final mean level of 0.74 nm. Loaded biosensors were first washed and transferred to wells containing seven APP-E2_JMR concentrations of a 2-fold dilution series (40 to 0.625 µM) in 1x kinetics buffer. Association and dissociation kinetics were recorded at least three times for 2.5 and 5 minutes at a shake speed of 1000 rpm, respectively. A second column of eight non-coated sensor tips and a 1x kinetics buffer well were used for double referencing of the raw data. Data were processed using Octet Data Analysis Software 7.0 (ForteBio) and were done in triplicate.

## Results

### The APP_695_-ectodomain consists of two folded domains

In order to test the anticipated multi-domain architecture of APP_695_ we expressed the entire ectodomain and subjected it to limited proteolysis by V8 endoprotease, trypsin, elastase and thermolysin. The proteases cleave hereby preferentially in regions of higher flexibility but not within compactly structured elements, thereby probing the folding-state of a given protein. The employed proteases gave similar results. The clearest picture was obtained when we incubated APP-Ecto with V8-protease. Potential cleavage sites were hereby distributed throughout the entire protein (Figure S1). Upon exposure to the protease, the protein is trimmed down to four stable fragments (Figure 1B). Identification of those fragments by Edman-Sequencing and MALDI-MS (Table 1, Figure S2-S5) shows that fragments two and four derive from the N-terminal part, whereas fragments one and three originate from the second half of the entire ectodomain indicating two rigidly folded domains. Further support of this general architecture comes from the existence of the two longer splicing forms APP_751_ and APP_770_ that contain in comparison to APP_695_ additional protein segments (the KPI-region and Ox-2) in between the acidic region and the E2-domain (Figure 1A). Because of its high structural homology to other Kunitz-type protease inhibitors [12], the KPI-region has been described as an independent folding unit. In line with the notion that sites of alternative splicing often correspond to structural domain boundaries [29], also the regions pre- and successing the KPI sequence in APP_751/770_ (and correspondingly the two halves of the ectodomain in APP_695_) are likely to represent structurally independent protein units. 

**Table 1:** Cleavage sites of limited proteolysis products.

Fragment^a^	N-terminal sequence (identified by Edman degradation)	Molecular weight (determined by mass spectrometry)	Deduced cleavage site^b^	Theoretical molecular weight	Deduced sequence^b^
1	VVRV	27.9 kDa	E(524)↓T(525)	27.9 kDa	V^286^VRV … SLTE^524^
2	MLEV^c^	23.7 kDa	E(227)↓(V228)	23.8 kDa	ML^18^EV … KVVE^227^
3	AMLN	16.0 kDa	E(524)↓T(525)	16.1 kDa	A^388^MLN … SLTE^524^
4	MLEV^c^	19.9 kDa	E(192)↓S(193)	20.0 kDa	ML^18^EV … LAEE^192^
5	STPD^c^	25.5 kDa	R(510)↓I(511)	25.5 kDa	S^295^TP … SEPR^510^
6	VEAML	15.9 kDa	R(510)↓I(511)^d^	14.9 kDa^d^	V^386^EA … SEPR^510^

^a^ numbers according to Figure 1

^b^ numbering according to APP_695_

^c^ sequences identified by Edman degradation correspond to the N-Terminus of the respective APP-constructs

^d^ According to the primary structure the next possible cleavage site is after R510, which is within the experimental error close to the observed molecular weight of 15.9 kDa.

To determine the exact boundaries of potentially folded and flexible segments within these two protein halves, limited proteolysis experiments were performed with the isolated APP-E1_ED_AcD and APP-E2_JMR constructs, corresponding to the respective complete N- and C-terminal protein segments (Figure 1A). In this way, we tremendously reduced the inherent complexity of the limited proteolysis experiments. 

**Figure 2:**
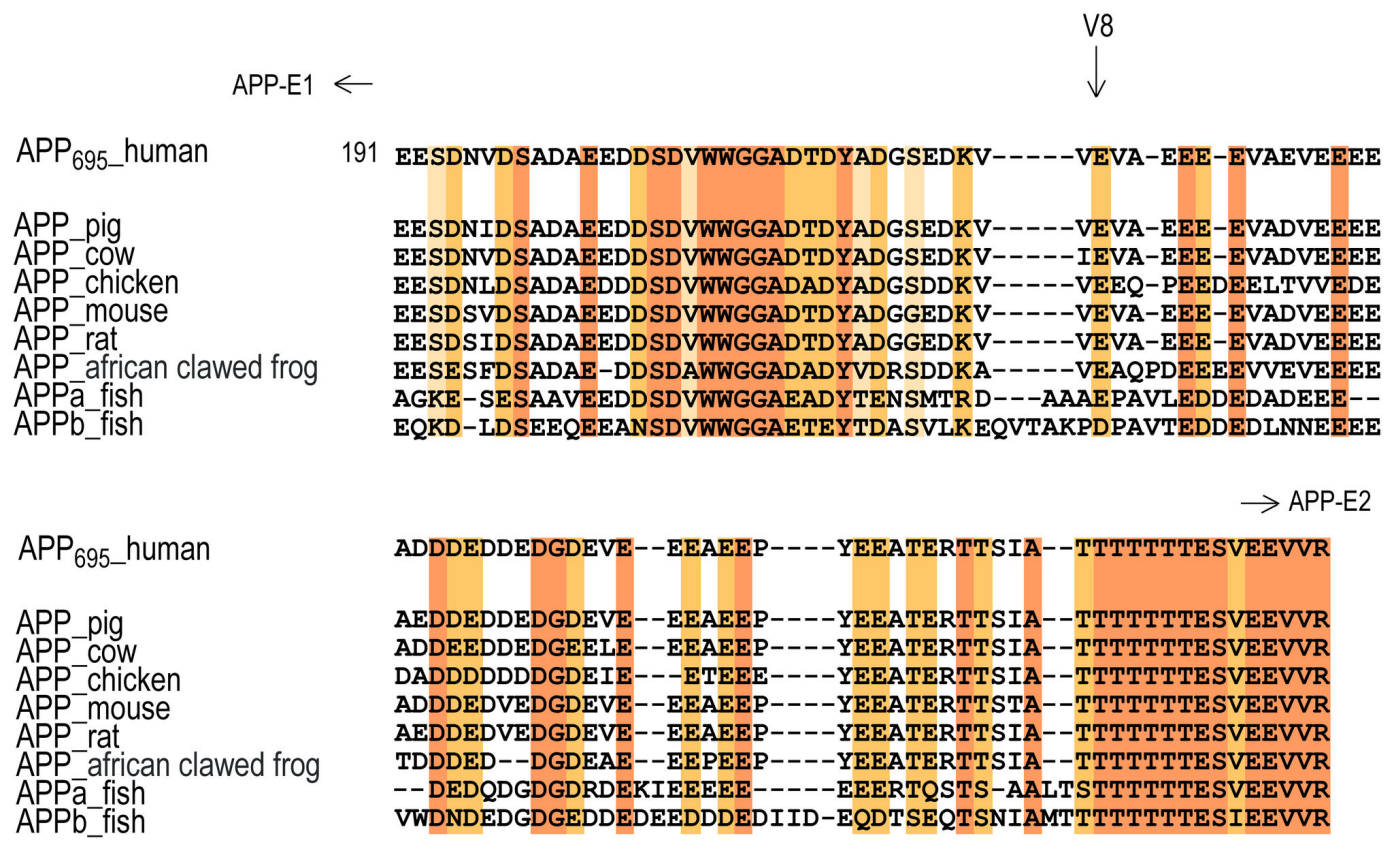
Sequence alignment of the acidic region among vertebrates. Fully conserved amino acids are highlighted on an orange background. The light yellow and darker yellow background indicate a fully conserved strong group and a conserved weaker group, respectively, according to ClustalX2.1 [28]. The cleavage site of the V8 protease is marked by a vertical arrow. The relative location of the E1- and the E2-domain are indicated by horizontal arrows.

Limited proteolysis of APP-E1_ED_AcD with V8‑protease resulted in two major fragments of ~24 kDa and ~20 kDa in size that remained stable after 24 h of digestion (Figure 1C). The exact N- and C-terminal boundaries of those fragments were determined by Edman sequencing and MALDI-MS (Table 1) and are identical to the fragments generated by limited proteolysis of APP-Ecto. Fragment two contains in comparison to fragment four an additional region of 35 amino acid residues at its C-terminus that is conserved among vertebrates (Figure 2) and is referred to hereafter as extension domain (ED). Interestingly, this fragment contains also phosphorylation sites [30] that could influence the physiological function of APP, and Vella et al. demonstrated recently that N-terminal fragments with a similar size are also generated in mouse and human brains [31]. Likewise, the incubation of APP-E2_JMR with trypsin results in two stable fragments of around 30 kDa and 20 kDa on SDS-PAGE (Figure 1D). The subsequent determination of its N- and C-terminal amino acid residues by Edman degradation and MALDI-MS (Table 1) showed that the longer fragment five corresponds to an N-terminal extension of fragment six. Only upon prolonged exposure to trypsin, this fragment is truncated to fragment six, suggesting some residual flexibility within the N-terminal region of APP-E2_JMR. Both fragments were already described by Dulubova et al. [32] and are similar to the fragments one and three generated upon exposure of the entire ectodomain to V8 protease. 

In summary, these results show that the APP-ectodomain contains two folded domains that are stable against proteolytic degradation. Interestingly, the termini of fragments four and five roughly correspond to the E1- and E2-domains that were initially identified based on homology between APP and members of APP-like family of proteins [6,7]. 

### The E1- and E2-domains represent regions of rigid fold

The protein segments corresponding to fragments two and four (referred to hereafter as APP-E1_ED and APP-E1, respectively) were cloned and purified for additional biochemical studies. In addition, we also investigated an already existing construct very similar to fragment five (APP-E2,[15]). The corresponding protein preparations were analyzed by gel permeation chromatography (GPC) coupled static light scattering (SLS) to determine both, the hydrodynamic radius based molecular weight (MW_rh_) and the absolute molecular weight (MW_SLS_) (Table 2). For tightly folded, spherical protein-molecules, both values should be similar, whereas one expects the MW_rh_ to be significantly higher than the MW_SLS_ if flexible segments are present. For all three constructs, the MW_SLS_ fits quite well to the respective theoretical molecular weights (MW_th_), showing that APP-E1, APP-E1_ED and APP-E2 are monomeric under the employed experimental conditions. The molecular weights of APP-E1 and APP-E2 determined by their retention volume in the GPC experiments (MW_rh_) are slightly larger than the theoretical molecular weights. The small difference is, however, readily explained by their non-spherical shape such that both APP-E1 and APP-E2 represent rigidly folded domains without flexible or extended linkers. This is also in line with the observation of these domains as major products in limited proteolysis (see above) and the fact that both domains were crystallized and their structures have been solved by X-ray crystallography [14,15,16,20].

**Table 2:** Apparent and absolute molecular weight of APP-constructs.

	MW_rh_ (kDa)	MW_SLS_ (kDa)	MW_th_ (kDa)
APP-E1	29^a^	22.9 ± 0.2^a^	21.7
APP-E1_ED	46	23.4 ± 2.5	25.6
APP-E1_ED_AcD	88	33.6 ± 1.9	32.3
APP-E2	27	27.0 ± 0.3	25.6
APP-E2_JMR	75	40.6 ± 0.5	40.1
APP-Ecto	178	72.2 ± 5.8	69.1

^a^ values taken from [20]

In contrast, the MW_rh_ measured by analytical gel filtration for APP-E1_ED is significantly higher than the absolute molecular weight indicating the presence of a flexible or extended linker region. We also observed repeatedly proteolytic degradation bands in between fragment two (APP-E1_ED) and four (APP-E1) during the preparation of APP-E1_ED (data not shown) further suggesting protease accessibility and hence flexibility of the protein segment at the C‑terminus of APP-E1_ED. 

### The acidic and juxtamembrane regions are flexible

Our limited proteolysis experiments revealed that the acidic and juxtamembrane regions are readily degraded indicating less secondary structure elements. To gain a deeper understanding about the structure of both regions, the respective molecular weights were first determined by GPC. Interestingly, APP-E1_ED_AcD and APP-E2_JMR show a significantly higher MW_rh_ than their theoretical molecular weight (Table 2). Correspondingly, they could either be dimeric in solution or they could contain a flexible section or extended linker that would result in an increased hydrodynamic radius. To further analyze this, GPC coupled SLS was performed, yielding their absolute molecular weights (MW_SLS_). These data are in excellent agreement with the theoretical molecular weights of the constructs, indicating that both proteins exist as monomer in solution. The difference between MW_rh_ and MW_SLS_ is, however, too large to be explained solely by the non-spherical shape of the respective fragments. Correspondingly, one has to conclude that both, APP-E1_ED_AcD and APP-E2_JMR contain flexible protrusions. As the isolated E1- and E2-domains correspond to rigidly folded domains, we conclude that both, the AcD and the JMR are predominantly flexible without strong secondary structure elements, which is also in excellent agreement with our results from limited proteolysis. In addition, CD experiments of APP-E1_ED_AcD and APP-E2_JMR confirm a higher amount of random coil in comparison to the rigidly folded E1- and E2-domains (Figure S6, Table S1). 

### E1- and E2-domain do not interact tightly

**Figure 3:**
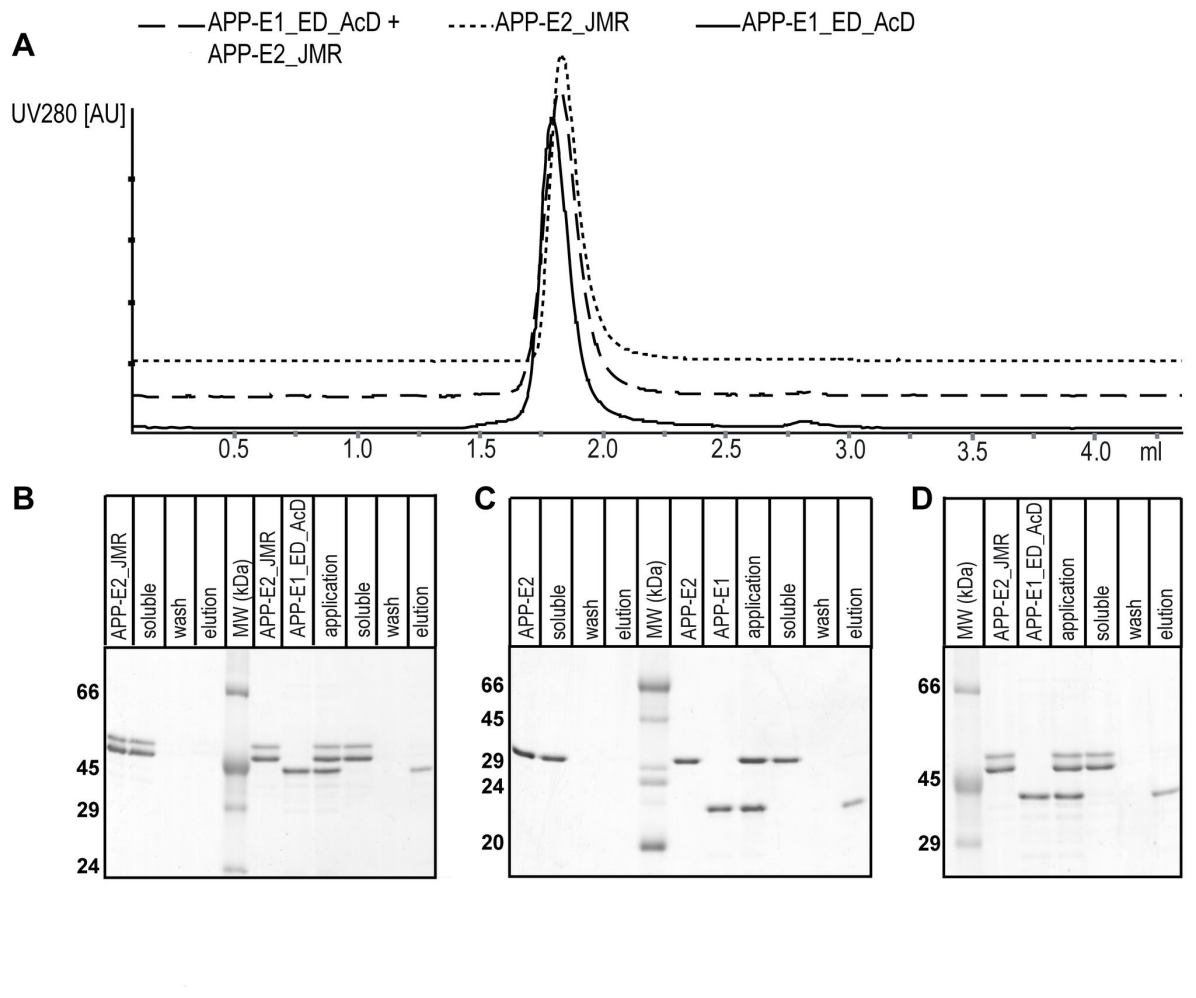
Interaction studies of the E1- and E2-domain. (a) Analytical gel filtration using the constructs APP-E1_ED_AcD and APP-E2_JMR. The UV-traces of the GPC analysis of the isolated and mixed proteins are given as indicated on top of the panel. For clarity the baseline is shifted arbitrarily. Upon mixing no increase of the molecular weight was observed, indicating the lack of a strong interaction between both constructs. (b) Pull-down assay with APP-E1_ED_AcD (His-tagged) and APP-E2_JMR (no His-tag) using Ni-NTA material. After centrifugation, APP-E2_JMR remains completely in the supernatant (soluble) and only His-tagged APP-E1_ED_AcD can be eluted. The first four lanes show that APP-E2_JMR (no His-tag) does not bind unspecifically to Ni-NTA (control). The sizes of the molecular weight (MW) marker bands are given on the left of the gel in kDa. (c) The pull-down assay with the constructs APP-E1 (His-tagged) and APP-E2 (no His-tag) demonstrates no interaction and shows that the flexible linker has no influence on a potential interaction of the domains. (d) Pull-down assay in the presence of low molecular weight heparin (10-12 sugar rings) indicates that heparin does not influence an interaction of APP-E1_ED_AcD and APP-E2_JMR.

To determine whether the ectodomain of APP is extended or whether the E1- and E2-domains interact with one another, analytical gel filtration was performed with the isolated as well as the mixed constructs APP-E1_ED_AcD and APP‑E2_JMR. The correct fold of the respective protein preparation was ensured by confirming their binding to heparin during purification and the analysis of their expected secondary structure in CD-spectroscopy (Figure S6). An interaction of both domains should lead to an increased apparent molecular weight compared to the respective individual constructs. The isolated constructs elute in Gaussian shaped peaks at 1.78 and 1.83 ml from the gel filtration column (Figure 3A). Upon mixing an intermediate retention volume of 1.82 ml is observed, showing that no strong interactions between both domains exist. This could be confirmed by respective pull-down assays (Figure 3B). APP-E2_JMR without His-tag is represented by a double band in SDS-PAGE although the N-Terminus determined by Edman-degradation and the C-Terminus (His-tag) is identical, since both bands interact with the His_5_ antibody and both bands shift in a SDS gel after His-tag removal. APP-E2_JMR without the tag does not bind to the NiNTA beads. If applied together with APP-E1_ED_AcD, it remains in the soluble fraction whereas only APP-E1_ED_AcD binds *via* its His-Tag to the NiNTA beads and elutes as isolated protein. To analyze whether the flexible regions influence a possible interaction, pull-down experiments were repeated using APP-E1 and APP-E2 (Figure 3C). Similar to the longer constructs also the rigidly folded domains were able to bind to heparin and were folded in CD-spectroscopy (Figure S6) demonstrating that the batch of purified protein is well folded. As expected from the longer constructs, also APP-E2 does not bind to the NiNTA beads, whereas the His-Tag containing APP-E1 binds and elutes as isolated protein, showing again no interaction between APP-E1 and APP-E2. This fits nicely to the very high apparent molecular weight determined for the whole APP-ectodomain in analytical gel filtration (Table 2). The absolute molecular weight measured by SLS is in good agreement with the theoretical molecular weight indicating that the construct is monomeric in solution under the experimental conditions. Correspondingly, the large difference between MW_rh_ and MW_SLS_ results from an extended non-spherical shape of the entire ectodomain in solution.

### The E1- and E2-domains do not interact in bio-layer interferometry experiments

**Figure 4:**
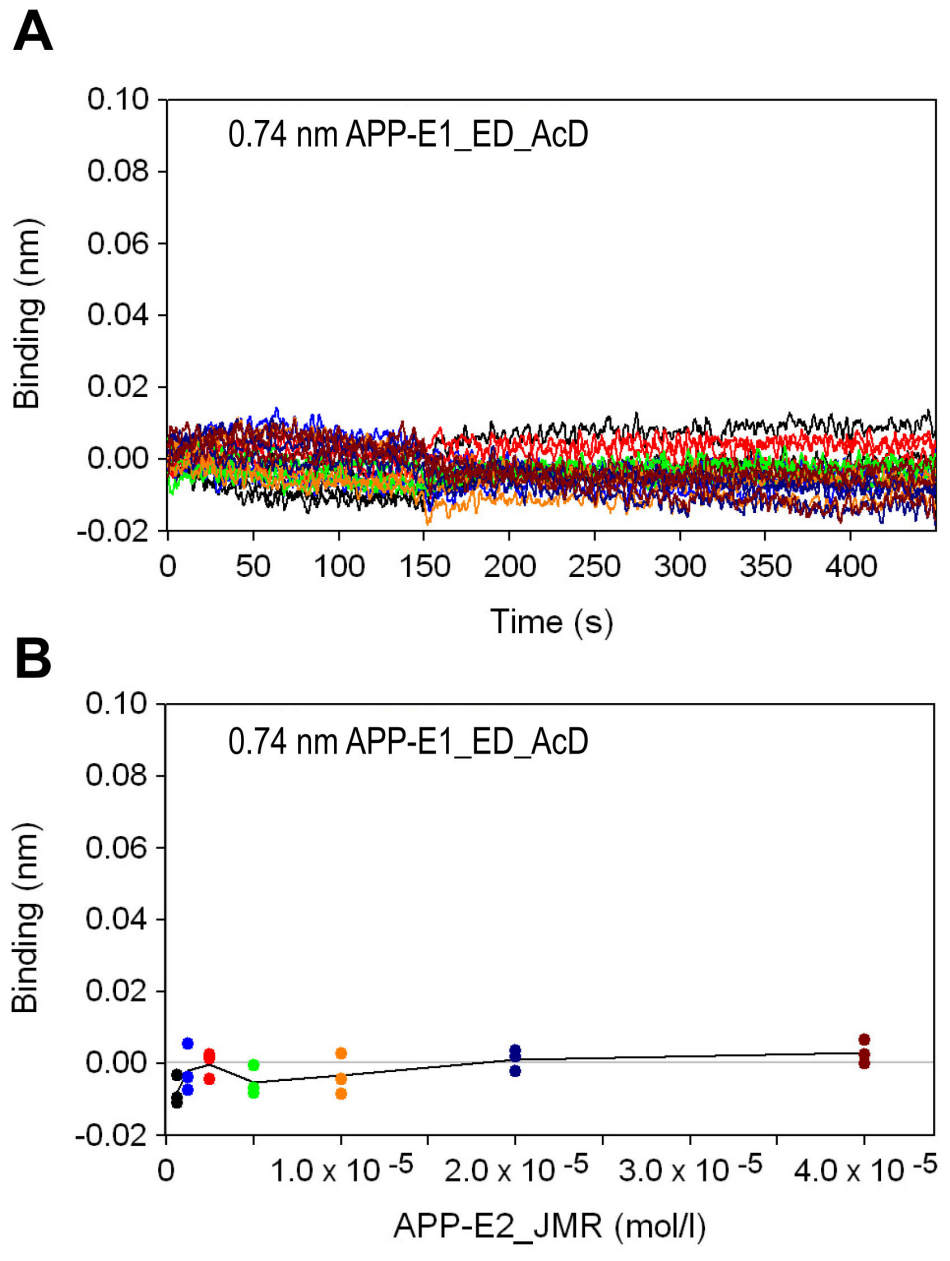
Real-time association and dissociation of APP-E2_JMR to immobilized APP-E1_ED_AcD. (a) Bio-layer interferometry sensorgrams of 40, 20, 10, 5, 2.5, 1.25, and 0.625 µM APP-E2_JMR binding to APP-E1_ED_AcD loaded on Streptavidin biosensors. Triplicate measurements were performed at each concentration in a 2-fold dilution series. (b) Plot of the concentration dependent APP-E2_JMR binding responses at the end of the association phase (150 seconds).

To analyze whether there is a potential weak interaction between the E1- and the E2-domain that might not have been detected in the above experiments, we performed interaction studies by bio-layer interferometry (BLI) [33]. In agreement with other methods, the assay demonstrated that the APP-E2_JMR domain does not bind to the APP-E1_ED_AcD domain with a measurable affinity, even at the highest concentration of 40 µM (Figure 4). This could be confirmed by surface plasmon resonance (SPR), where we were not able to detect a specific binding reaction between APP-E1_ED_AcD and APP_E2_JMR as well as between APP-E1 and APP-E2 (M. C. Mayer and G. Multhaup, personal communication).

### Heparin does not support an interaction between the E1- and E2-domain

Both, the E1- and the E2-domains bind to heparin [34,35]. Therefore, we hypothesized that heparin might influence the interaction between both domains. To investigate this, we performed pull-down assays in the presence of short and long heparin-chains (10-12 and ~55 sugar rings). As seen before, APP-E2_JMR remains completely in the soluble fraction, whereas only isolated His-tag-containing APP-E1_ED_AcD binds to the Ni-NTA material (Figure 3D and data not shown). This demonstrates that heparin does not strongly influence an intramolecular interaction of APP-E1 and APP-E2.

## Discussion

The amyloid precursor protein (APP) and its proteolytic processing by the α-, β- and γ-secretases are critically involved in the development of Alzheimer’s disease, and highly resolved structural data were obtained by protein crystallography and NMR-spectroscopy for several segments of this multi-domain protein. However, until now, rather little is known about its overall structure, its exact domain architecture, the structure and the extent of flexible linker segments and the possible interaction of its constituting domains. Such knowledge is, however, indispensable to interpret functional data of this protein within the correct three-dimensional, structural framework. Our limited proteolysis experiments together with GPC coupled SLS experimentally demonstrate that the large ectodomain of APP_695_ contains two rigidly folded regions – the E1- and E2-domains. Correspondingly, the longer splicing forms APP_751_ and APP_770_ contain the KPI-domain as third rigidly folded building block [12,13] inserted in-between E1 and E2. We mapped the boundaries of these two domains to be most likely Leu18-Ala190 (E1-domain) and Ser295-Asp500 (E2-domain), respectively (APP_695_-numbering), which fits well to the regions of defined three-dimensional structure seen in the respective structures (Leu28-Ala190 for E1-domain [20] and His313-Gln492 [14] as well as Thr296-Leu491 [16] for E2-domain). This domain definition is in agreement with and further refines the extent of the predicted E1- and E2-domains, which were originally assigned based on homology [7]. Interestingly, the structures of the GFLD and the CuBD within the E1-domain were initially reported as independent entities [9,10], and SAXS-studies showed controversial results with respect to their interaction [25,26]. Using protein crystallography, limited proteolysis and GPC coupled SLS we previously found that they interact tightly at slightly acidic pH, constituting together the E1-domain [20]. Our present study shows a tight interaction of the GFLD and the CuBD also at neutral pH. 

The E1-domain is followed by a protein segment exhibiting a high percentage of acidic amino acid residues (56 %) that is typically referred to as the acidic domain (AcD) and connects the E1- and the E2-domain. Such a negatively charged region is unlikely to form a tightly folded structural entity. Sequence alignments show, however, a certain degree of conservation of this region between vertebrates (Figure S1), suggesting functional relevance. We hence analyzed its contribution to the overall structure of APP and found that it confers flexibility, evidenced by its rapid degradation in limited proteolysis and by a dramatically increased hydrodynamic radius of the APP-E1_ED_AcD construct as compared to the rigidly folded E1-domain. This finding is in excellent agreement with the observation that this region is easily degraded *in vitro* [9]. Directly C-terminal to the E1-domain, a region of ~30 amino acid residues shows a high degree of conservation, and might represent another folded segment as it does not contain primarily acidic residues. Although we identified a respective band in limited proteolysis, subsequent experiments clearly showed flexibility within this section. However, the high degree of conservation and the presence of phosphorylation sites within this segment [30] strongly suggest a role for the physiologic function of APP. This was further demonstrated by the identification of the corresponding N-terminal APP-fragments in human and mouse brains [31]. 

**Figure 5:**
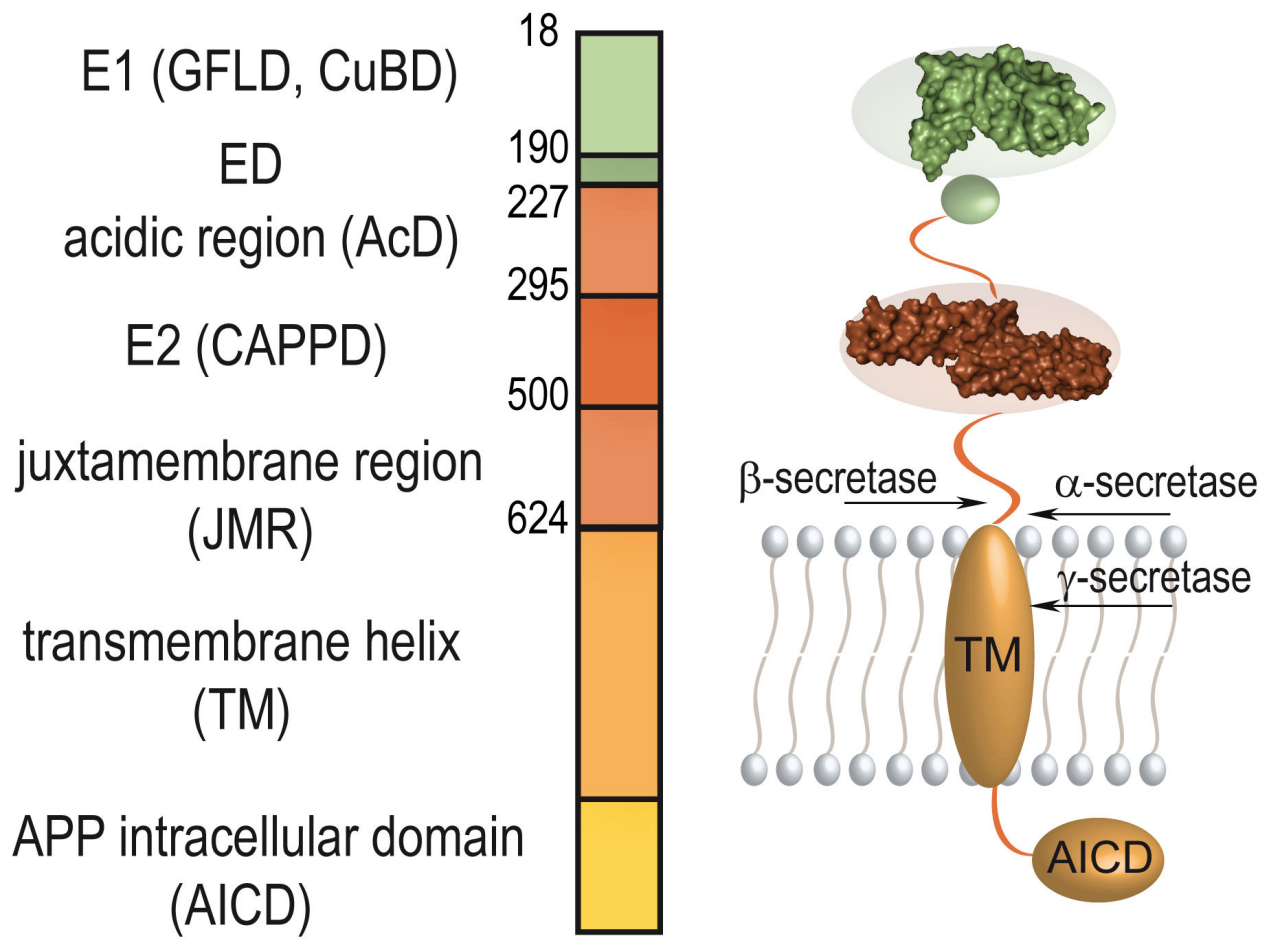
Overall structure of the amyloid precursor protein. The APP_695_ ectodomain consists of exactly two folded domains: the E1- (AA18-190) and E2-domain (AA295-500). The acidic domain (AcD) in between both domains is as flexible as the juxtamembrane region (JMR). Furthermore, the E1- and E2-domains do not interact with one another tightly, leading to an extended overall conformation. The extension domain (ED, AA191-227) is located in-between E1 and AcD as additional region of partial structure. The two rigidly folded domains of the ectodomain are shown as surface presentation based on their respective crystal structures ([20] PDB-ID: 3ktm [16]; PDB-ID: 3nyl).

The folded E2-domain is connected to the single transmembrane helix of APP by the juxtamembrane region (JMR), which represents a second linker and shows less conservation compared to the E1- and E2-domains (Figure S1). Using GPC coupled SLS and limited proteolysis we could experimentally demonstrate that also this region is flexible. Thus, the large ectodomain of APP represents a rather flexible arrangement of its rigid constituents, the E1- and E2-domains that are connected to one another and to the single transmembrane helix like beads on a string by the flexible connections AcD and JMR, respectively (Figure 5). This overall arrangement is further supported by the rather large hydrodynamic radius exhibited by the whole ectodomain, which we could show by SLS-studies to be completely monomeric under experimental conditions. The observed high MW_rh_ might hereby easily be interpreted as indicating dimerization of the APP-ectodomain, underscoring the importance of determining the absolute molecular weight. 

Interestingly, the E1- and E2-domains of APP are highly conserved between this protein and the APP-like proteins [7,8]. Often such conserved domains are tightly correlated with specific functions [36], suggesting more than one physiologic role for the full-length protein. Several such possible functions have been described for the two rigidly folded domains of APP. In contrast, functions of the AcD and the JMR are rather unknown. Prompted by controversial SAXS-results [25,26], we investigated whether the E1- and E2-domains might interact directly, potentially induced and regulated by the AcD and/or the JMR. Using analytical gel filtration experiments and pull-down assays as well as BLI and SPR we could show that neither the folded domains E1 and E2 nor the extended constructs APP-E1_ED_AcD and APP-E2_JMR do interact with one another. Also different length heparins, that were shown to bind to both folded domains [20,25,37,38,39], did not promote such a direct interaction. These data are in excellent agreement with the hydrodynamic radius determined here for the entire ectodomain (APP-Ecto) that fits to a molecule of elongated shape (Table 2). 

Interestingly, the cleavage sites of the β- and the α-secretase, discriminating between the amyloidogenic and the non-amyloidogenic processing of APP, are located at the C-terminus of the JMR next to the single transmembrane helix of APP. The cleavage of Notch, a cell-surface receptor that is processed similar to APP by a ADAM-type metalloprotease at site S2 and afterwards by γ-secretase, is regulated by a conformational change [40]. The high degree of flexibility and hence the absence of any tight folding inside the APP-JMR suggest a different regulation mechanism. Correspondingly, it has been shown that the exposition of APP to the different secretases is largely influenced by its trafficking. Protein-protein interaction with F-Spondin and SorlA represent regulatory events of its trafficking and its processing by proteolytic enzymes [41,42]. Nevertheless, the insertion of native APP in the lipophilic environment of the membrane and the presence of its single transmembrane helix probably also influences the structure of other parts of the protein. It is therefore important to consider the above described results with respect to the recently determined structure of the APP-fragment and γ-secretase substrate C99 [17]. Its membrane insertion and the identification of a closeby cholesterol binding site will probably affect cleavage by α-secretase. The β-secretase cleavage site after Met596 is located 16 amino acid residues further N-terminal (at the N-terminus of C99) and was completely unstructured in line with the data presented here (Figure 5).

In conclusion, we could experimentally prove that the multi-domain protein APP has an extended overall topology and consists of two tightly folded segments, the E1- and E2-domains. They are linked to one another by the flexible protein segment AcD, but do not show any direct protein-protein interaction, neither in the presence nor in the absence of heparin. This assembly is connected to the membrane by the again flexible JMR, containing the α- and β-secretase cleavage sites. This work represents a thorough experimental characterization of the overall structure of APP by different methods and will prove very valuable to gain a deeper understanding about the structure-function-relationship of the protein in the future. Several described functions of APP were originally identified with isolated peptides. Only the native arrangement of functional amino acids in space, however, can accommodate their true physiologic function, underlining the importance of the present investigation. On the next level of organization and interaction, APP is probably subject to a number of oligomerization events and additional protein-protein-interactions. Those must build, however, on the here identified three-dimensional structure of the isolated, monomeric protein. 

## Supporting Information

Figure S1 **Sequence alignment of the APP ectodomain among vertebrates.** Fully conserved amino acids are highlighted on an orange background. The light yellow and darker yellow background indicate a fully conserved strong group and a conserved weaker group, respectively, according to ClustalX2.1. Potential cleavage sites of trypsin are marked by a vertical arrow. The black and grey (100-300 times slower) arrowheads indicate potential V8 protease cleavage sites.
(TIF)

Figure S2 **MS analysis of fragment one, two and four.**
(TIF)

Figure S3 **MS analysis of fragment five.**
(TIF)

Figure S4 **MS analysis of fragment six.**
(TIF)

Figure S5 **MS analysis of fragment two, three and four.**
(TIF)

Figure S6 **CD-Spectroscopy.** APP-E1_ED_AcD and APP-E2_JMR indicated by a blue line show a higher amount of random coil than the proteolysis products APP-E1 and APP-E2 that are shown as a green line.
(TIF)

Table S1 **Secondary structure contents measured by CD-spectroscopy.**
(DOC)
