# Supplementary figures and images for: Analysis of the Overall Structure of the Multi-Domain Amyloid Precursor Protein (APP)
Source: PLoS One. 2013 Dec 4;8(12):e81926. doi: 10.1371/journal.pone.0081926 (PMC3852973; doi:10.1371/journal.pone.0081926)

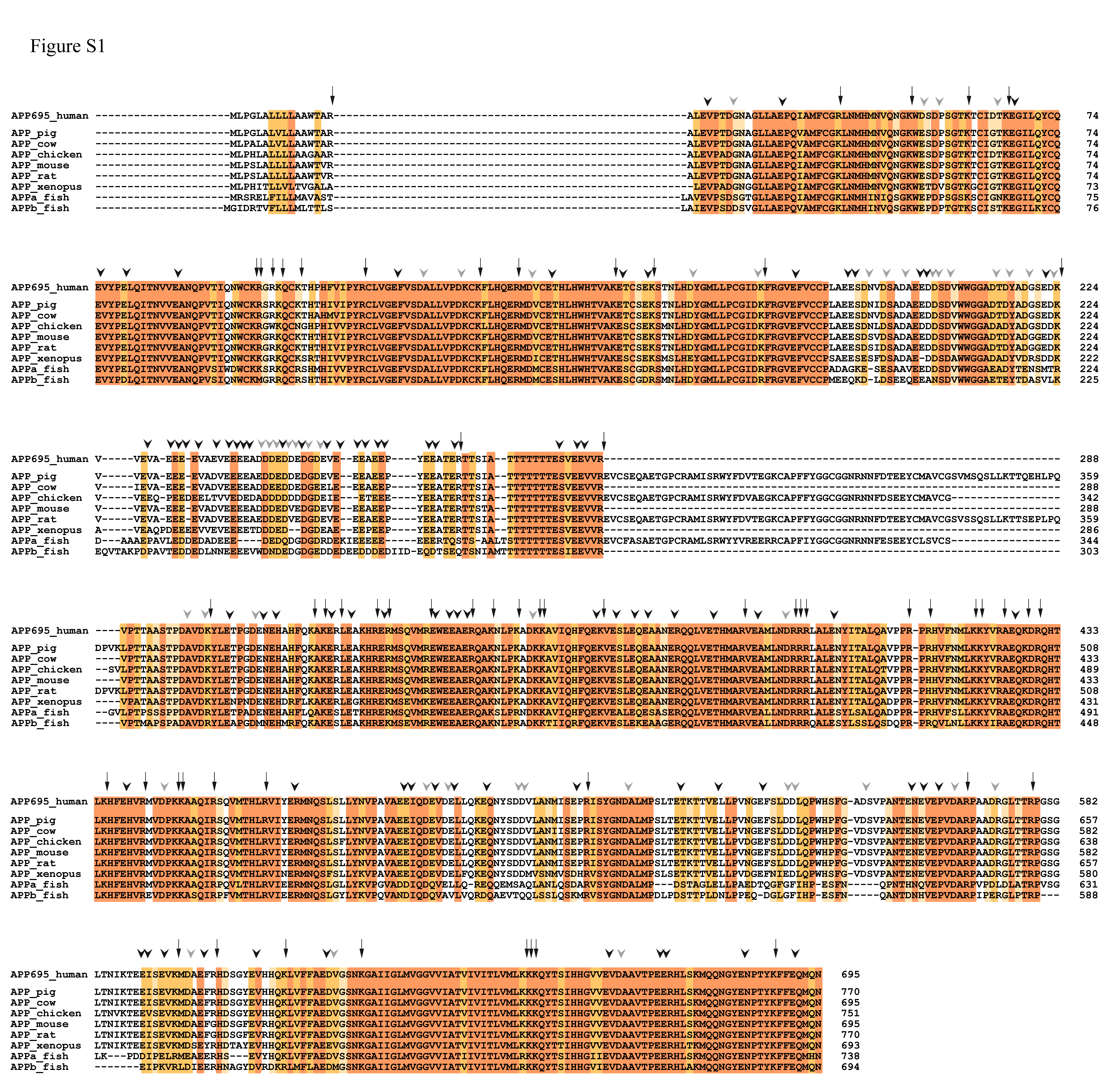

Supplement: Figure S1 — Sequence alignment of the APP ectodomain among vertebrates. Fully conserved amino acids are highlighted on an orange background. The light yellow and darker yellow background indicate a fully conserved strong group and a conserved weaker group, respectively, according to ClustalX2.1. Potential cleavage sites of trypsin are marked by a vertical arrow. The black and grey (100-300 times slower) arrowheads indicate potential V8 protease cleavage sites. (TIF) [file pone.0081926.s001.tif]

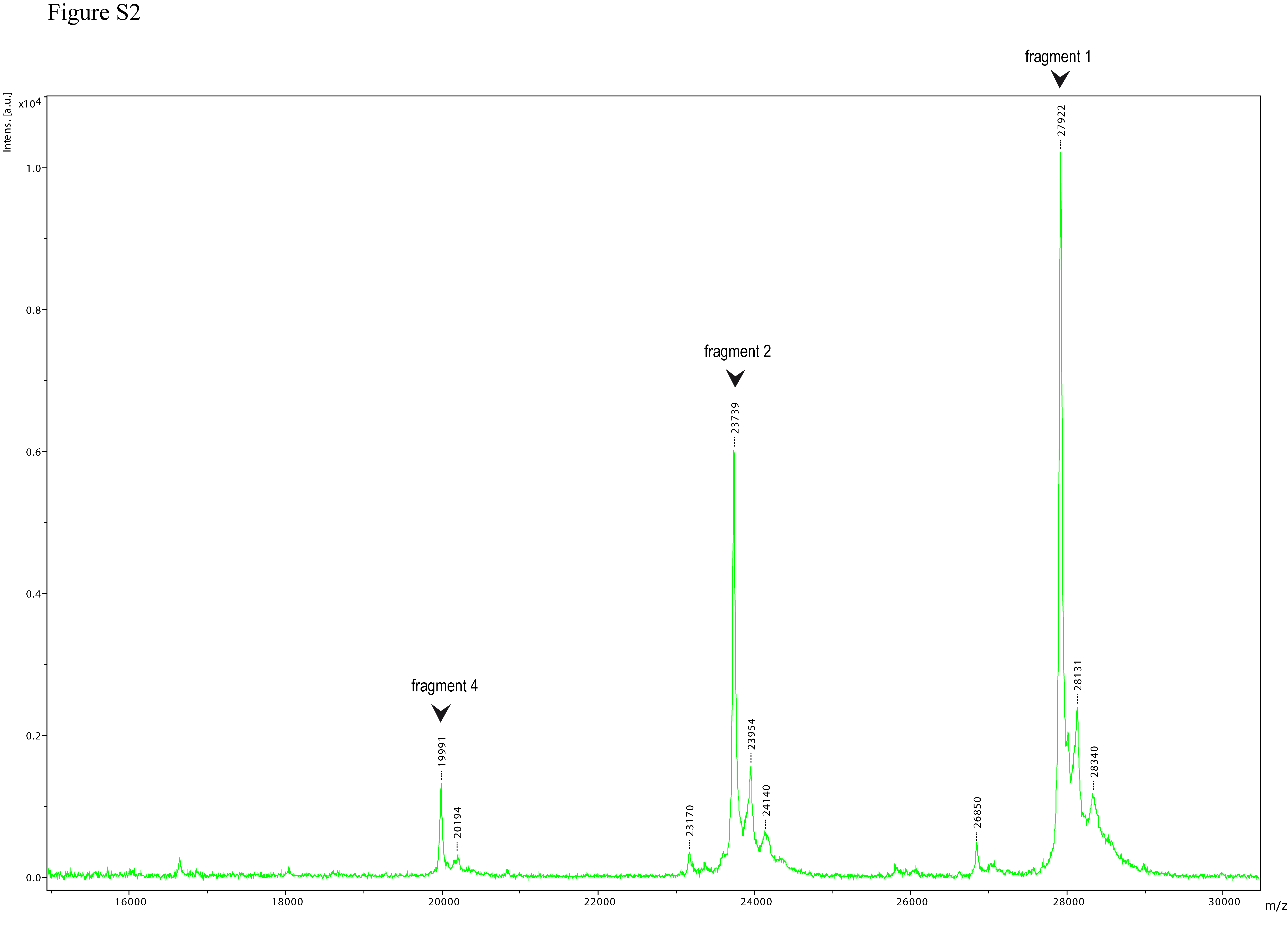

Supplement: Figure S2 — MS analysis of fragment one, two and four. (TIF) [file pone.0081926.s002.tif]

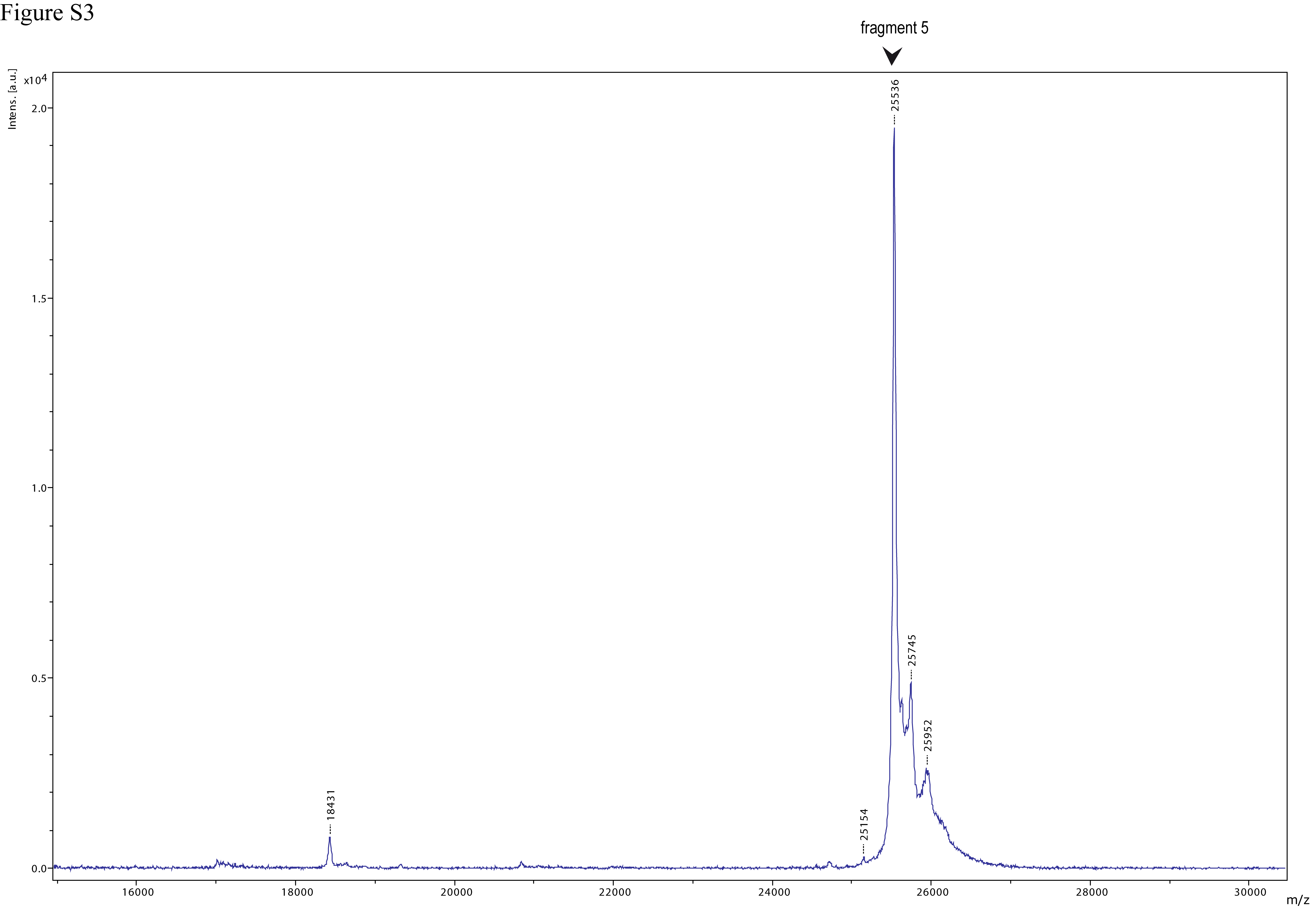

Supplement: Figure S3 — MS analysis of fragment five. (TIF) [file pone.0081926.s003.tif]

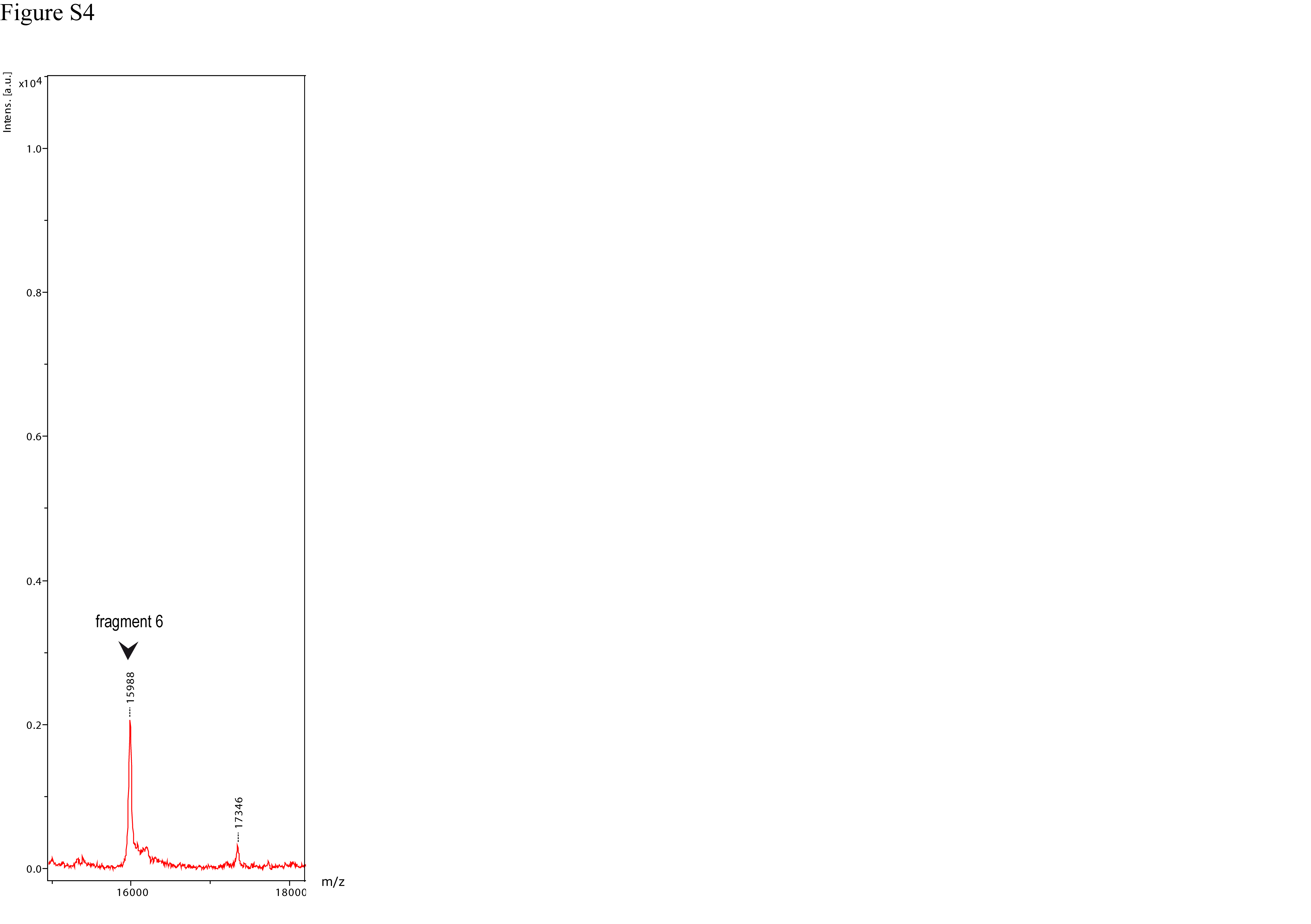

Supplement: Figure S4 — MS analysis of fragment six. (TIF) [file pone.0081926.s004.tif]

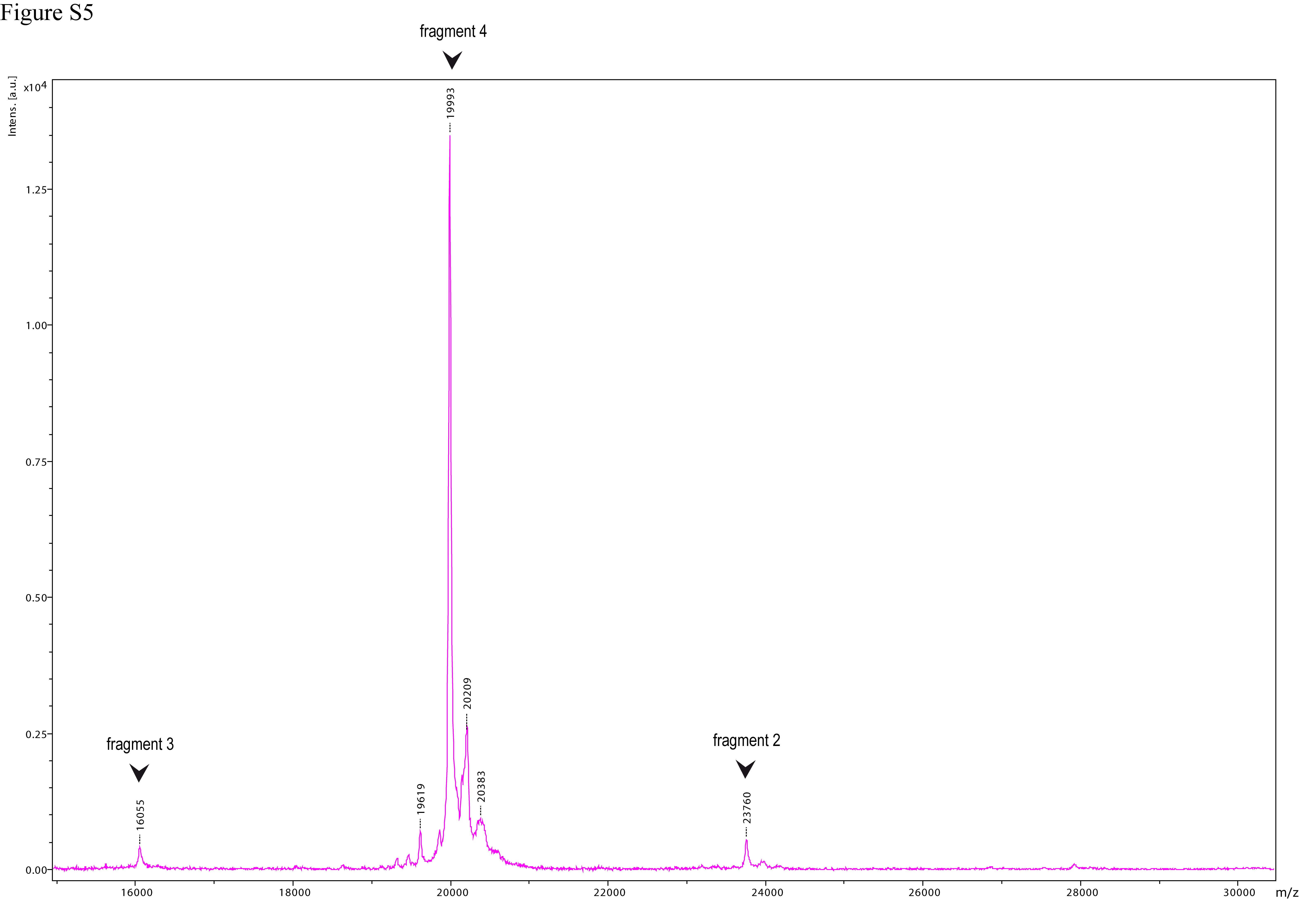

Supplement: Figure S5 — MS analysis of fragment two, three and four. (TIF) [file pone.0081926.s005.tif]

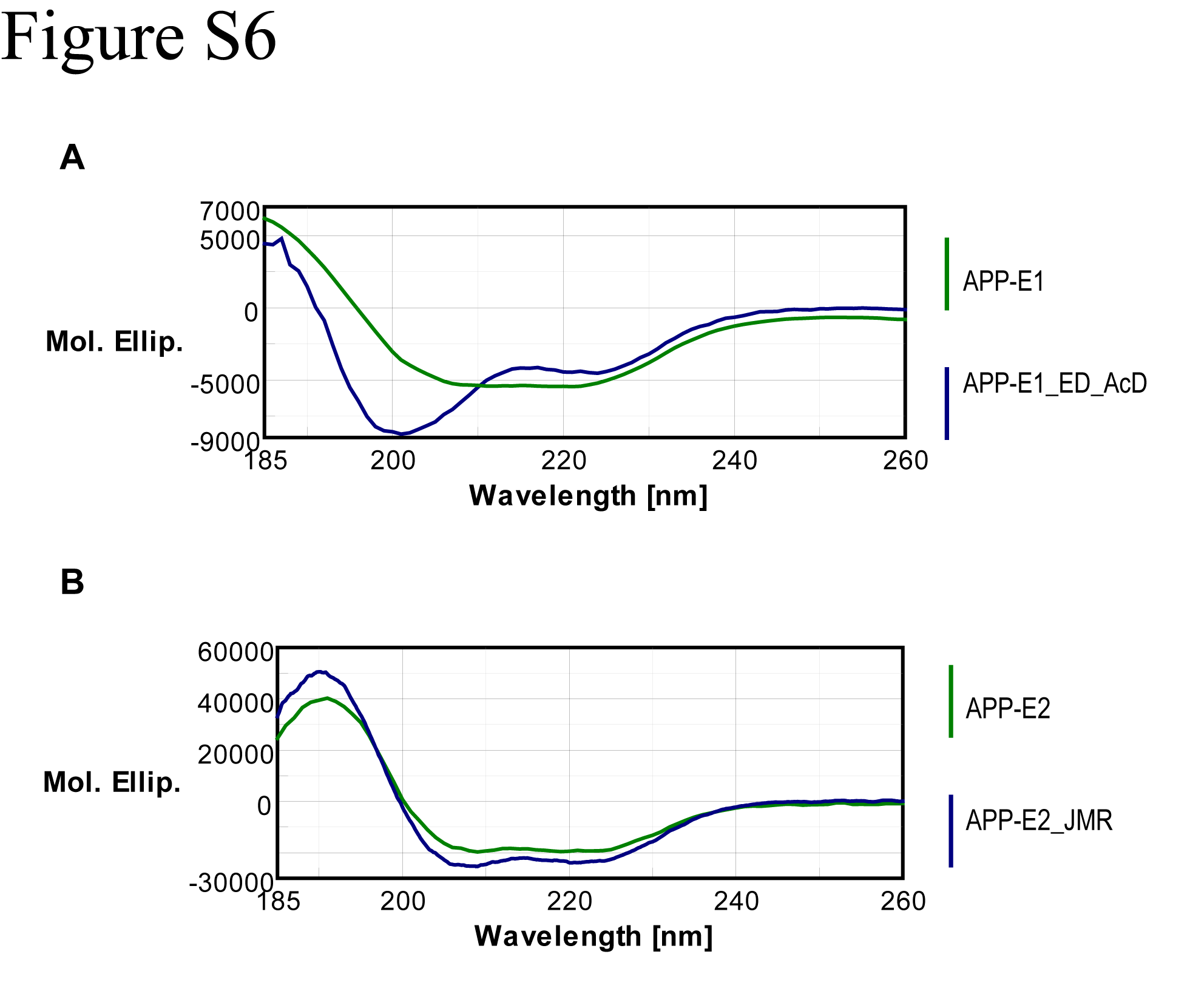

Supplement: Figure S6 — CD-Spectroscopy. APP-E1_ED_AcD and APP-E2_JMR indicated by a blue line show a higher amount of random coil than the proteolysis products APP-E1 and APP-E2 that are shown as a green line. (TIF) [file pone.0081926.s006.tif]
