# Supplementary material for: Analysis of the Overall Structure of the Multi-Domain Amyloid Precursor Protein (APP)
Source: PLoS One. 2013 Dec 4;8(12):e81926. doi: 10.1371/journal.pone.0081926 (PMC3852973; doi:10.1371/journal.pone.0081926)
Supplement: Table S1 — Secondary structure contents measured by CD-spectroscopy. (DOC) [file pone.0081926.s007.doc]

Table S1: Secondary structure contents measured by CD-spectroscopy

|  | APP-E1 | APP-E1_E1_AcD | APP-E2 | APP-E2_JMR |
| --- | --- | --- | --- | --- |
| α-helix | 12 % | 10 % | 77 % | 57 % |
| Antiparallel β‑sheet | 30 % | 25 % | 1 % | 0 % |
| Parallel β-sheet | 5 % | 4 % | 2 % | 7 % |
| β-turn | 20 % | 24 % | 7 % | 14 % |
| Random coil | 34 % | 38 % | 9 % | 23 % |
| Sum | 101 % | 101 % | 96 % | 101 % |
